# Mediastinal NUT Carcinoma With Raised Serum Alpha-Fetoprotein Mimicking a Malignant Germ Cell Tumor: Suspicion Raised Due to Negative Serum miR-371a-3p Levels

**DOI:** 10.1177/10935266251335391

**Published:** 2025-04-25

**Authors:** Sheng-Yuan Kan, Cinzia G. Scarpini, Dawn Ward, Ben Fleming, Heok K. Cheow, Ibrahim Jalloh, John A. Tadross, James Watkins, Thomas Roberts, Jamie Trotman, Patrick Tarpey, Nicholas Coleman, C. Elizabeth Hook, Charlotte Burns, Claire Trayers, Matthew J. Murray

**Affiliations:** 1 Department of Histopathology, Cambridge University Hospitals NHS Foundation Trust, Cambridge, UK; 2 Department of Pathology, University of Cambridge, Cambridge, UK; 3 Department of Radiology, Cambridge University Hospitals NHS Foundation Trust, Cambridge, UK; 4 Department of Neurosurgery, Cambridge University Hospitals NHS Foundation Trust, Cambridge, UK; 5 East Genomic Laboratory Hub (GLH) Genetics Laboratory, Cambridge University Hospitals NHS Foundation Trust, Cambridge, UK; 6 Medical Research Council Metabolic Diseases Unit, Institute of Metabolic Science Metabolic Research Laboratories, University of Cambridge, Cambridge, UK; 7 Department of Paediatric Haematology and Oncology, Cambridge University Hospitals NHS Foundation Trust, Cambridge, UK

**Keywords:** alpha-fetoprotein, *BRD4::NUTM1* gene fusion, germ cell tumor, miRNA, NUT carcinoma, serum miR-371a-3p testing, whole genome sequencing

## Abstract

NUT carcinoma is challenging to diagnose and may mimic a germ cell tumor (GCT) due to raised serum alpha-fetoprotein (AFP). A 15-year-old patient presented with back pain and cough. Investigation revealed a mediastinal mass and multiple bone metastases. Serum AFP was highly elevated, consistent with a metastatic malignant nonseminomatous GCT. Aggressive chemotherapy was initiated with initial response, unfortunately not sustained. Diagnostic biopsy showed undifferentiated tumor cells with weak GCT immunophenotype but was ultimately non-diagnostic. Serum miR-371a-3p levels, highly sensitive/specific for malignant GCTs, were negative casting diagnostic suspicion. Routine use of agnostic molecular investigations, including whole genome sequencing, identified a chromosome 15:19 translocation, with *BRD4::NUTM1* gene fusion on RNA sequencing, confirming NUT carcinoma. Subsequent NUTM1 immunohistochemistry was positive. A high index of clinical suspicion is required for non-pathologically/molecularly confirmed diagnoses. Serum miR-371a-3p quantification ruled out malignant GCT and routine agnostic molecular studies identified the correct diagnosis; a low threshold for NUTM1 immunohistochemistry is thus recommended.

## Introduction

The *NUTM1* gene is a fusion partner in the pathogenesis of aggressive and poorly-differentiated neoplasms, including NUT carcinoma. NUT carcinoma arise predominantly in the thorax (51%) and head/neck (41%), with equal sex distribution and a young median age of diagnosis of ~23 years.^1^ The formal incidence/prevalence of NUT carcinoma is unknown, with only ~300 cases reported in the literature.^2^ Histologically, NUT carcinoma has non-specific features, with sheets of typically small, undifferentiated, and monomorphic cells and features of mitosis, necrosis, acute inflammatory cells typically present^3^; some display foci of keratinization. Outcomes for patients with NUT carcinoma are very poor, with a median overall survival of just 6.5 months, despite treatment with intensive chemotherapy.^1^ A recent systematic review re-emphasized poor outcomes for thoracic/mediastinal cases.^4^

In contrast, germ cell tumors (GCTs) account for 10–20% of all mediastinal neoplasms, with incidence also peaking in young adulthood like NUT carcinoma. Histological characteristics of GCT are similar regardless of site, but mediastinal GCTs are classified as poor-risk.^5,6^ These tumors present as seminoma and nonseminomatous GCTs (NSGCTs), as well as teratoma, and are all typically characterized by morphology and specific patterns of immunohistochemistry. The serum tumor markers alpha-fetoprotein (AFP) and human chorionic gonadotrophin (HCG) assist in GCT diagnosis/monitoring.^7^ In patients with typical clinical presentation and radiology and raised AFP/HCG markers, treatment can be initiated for a malignant GCT without recourse to biopsy for those with tumors in sites such as the mediastinum, which are often associated with respiratory compromise and which may make anesthesia and biopsy challenging.^8^ However, AFP/HCG markers are not necessarily specific for GCT,^7^ causing diagnostic challenges. Of note, all malignant GCTs are characterized by universal overexpression of microRNAs (miRNAs) from the miR-371–373 and miR-302/367 clusters^9,10^ and our team were the first to identify that these miRNAs represented novel serum tumor markers.^11^ Since that time, serum miR-371a-3p (from the miR-371–373 cluster) quantification has been shown to be a very highly sensitive and specific marker for malignant GCTs,^12-14^ with the potential to overcome the lack of specificity of AFP.

Here we report a patient presenting with a mediastinal mass and highly raised serum AFP, initially diagnosed as a malignant GCT. However, diagnostic serum miR-371a-3p levels were negative, casting doubt on the diagnosis. Accordingly, multiple biopsies were taken, all challenging to interpret. Subsequent molecular testing including whole genome (WGS) and RNA sequencing identified a *BRD4::NUTM1* gene fusion diagnostic of NUT carcinoma. The case highlights the clinical utility of serum miRNA testing and agnostic routine molecular testing for cancer patients, as well as the need for maintaining a high index of suspicion to challenge putative diagnoses which have not been histologically/molecularly confirmed.

## Case Report

**Figure 1.**
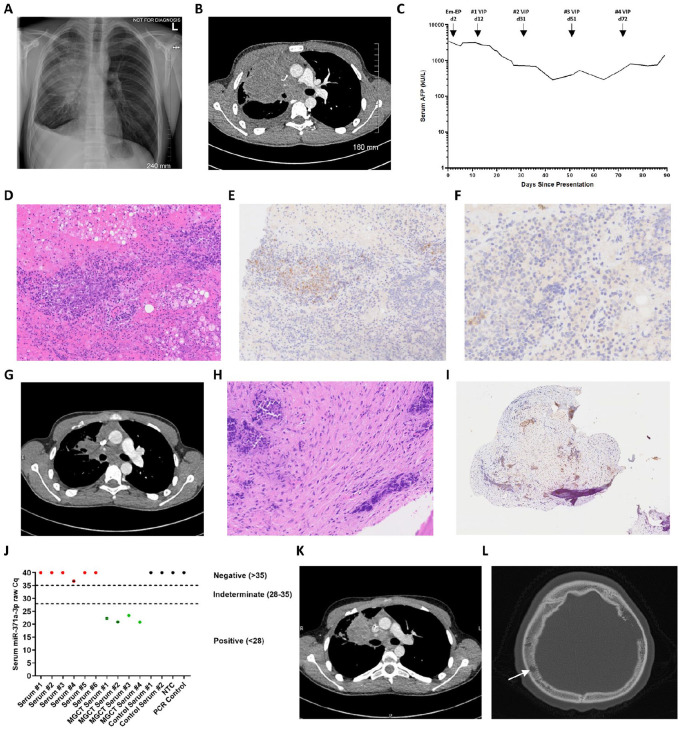
Representative biochemical, radiological, and pathological features of the NUT carcinoma case. (A) Chest X-ray at presentation revealing right-sided pleural effusion and mediastinal mass. (B) Representative CT scan at presentation showing large right thoracic/anterior mediastinal soft tissue mass causing compression of the right main bronchus. (C) Serial serum AFP levels from diagnosis through treatment. (D-F) Pathology of the mediastinal mass biopsy: (D) Representative H&E staining of the mediastinal mass biopsy, showing lung parenchyma with focal hemorrhage, acute inflammation, and abundant foamy macrophages. Occasional clusters of lesional cells were seen, mostly perivascular in location, which had small nuclei with uniform chromatin and a small rim of clear cytoplasm; (E and F) Immunohistochemistry showing the lesional cells to stain weakly positive for (E) SALL4 (surrounding vessels) and (F) Glypican-3. The cells also displayed weak positivity for CD99. They did not express AFP, CD30, PLAP, TdT, CD79a, CD3, Desmin, or CD117. INI-1 expression was retained. (G) CT reassessment showing a substantial decrease in the size of the anterior mediastinal mass with improvements in the caliber of the right main bronchus. (H and I) Pathology of the biopsy of the parietal bone lesion: (H) H&E staining showing fragments of bone with abundant necrosis and fibrosis within the medullary cavity. Scattered within the fibrosis were crushed cells with high nuclear:cytoplasmic ratios; these cells had pleomorphic, hyperchromatic nuclei and a rim of clear cytoplasm; (I) Immunohistochemistry showing weak AFP positivity. In addition, weak SALL4, patchy CD99, patchy CD117, diffuse Bcl-2, and focal EMA positivity was identified. CD3, CD79a, CD30, PLAP, Desmin, MyoD1, Glypican-3, OCT3/4, and WT1 were negative. There was no evidence of EWSR1 or SS18 gene rearrangement by FISH. Appearances were felt to be most in keeping with a NSGCT subtype of malignant GCT. However, a definitive diagnosis was not possible due to the small number of malignant cells and challenging morphology. Isochromosome 12p FISH testing was not possible due to the tiny amount of available biopsy tissue available and considerable crush artefact. (J) Serum miR-371a-3p results, revealing multiple negative results from serum samples at patient presentation, along with appropriate positive and negative controls. Negative results are raw Cq miR-371a-3p of ≥35; positive results are raw Cq <28, as described.^15^ (K and L) Representative images from CT scans undertaken at the time of clinical progression, (K) showing a substantial increase in the size of the anterior mediastinal mass with heterogeneous attenuation consistent with patchy necrosis, and (L) axial bone reconstruction demonstrating an increase in bony metastatic disease, with a large lytic lesion within the left parietal bone and an additional smaller lytic lesion within the right parietal bone (arrow).

A 15-year-old previously well male presented with a 3-week history of worsening back pain and 2 months of cough, weight loss, and fatigue. Examination revealed right-sided pleural effusion. Chest X-ray confirmed the effusion and identified a mediastinal mass (Figure 1(a)), confirmed by CT scan (Figure 1(b)), and multiple vertebral bony metastases, initially felt to be most consistent with metastatic sarcoma. Given respiratory compromise and anesthesia risk, awake, ultrasound-guided biopsy of the mediastinal mass was planned. Whilst arranging this, the serum AFP resulted at 3438 kU/L (reference range 0–7 kU/L; Figure 1(c)), potentially consistent with a metastatic mediastinal malignant NSGCT [poor-risk group as per the International Germ Cell Consensus Classification (IGCCC).^5,6^ Accordingly, the need for biopsy was carefully re-discussed. Given the pattern of metastatic disease (multiple bone lesions in the absence of pulmonary lesions) and discrepancy between the substantial tumor burden and comparatively modestly elevated AFP, the decision was made to proceed. CT head to complete staging demonstrated a left parietal bone metastasis but no parenchymal brain metastases. Given the extent of disease, emergency 2-day “EP” (cisplatin 20 mg/m^2^ and etoposide 100 mg/m^2^/day) was delivered on day 2/3 (d2/d3), to gently reduce tumor bulk and reduce risk of acute toxicities, prior to starting definitive treatment, as described.^8^

The mediastinal biopsy was paucicellular, showing lung parenchyma with focal hemorrhage, acute inflammation, and abundant foamy macrophages. Occasional clusters of lesional cells were seen, mostly perivascular in location, which had small nuclei with uniform chromatin and a small rim of clear cytoplasm (Figure 1(d)). Immunohistochemistry showing the lesional cells to stain weakly positive for SALL4 (surrounding vessels; Figure 1(e)) and Glypican-3 (Figure 1(f)). The cells also displayed weak positivity for CD99. They did not express AFP, CD30, PLAP, TdT, CD79a, CD3, Desmin, or CD117. INI-1 expression was retained. Ultimately, the biopsy was non-diagnostic. No suitable material for any further molecular testing was available, including WGS. After an initial very early fall of serum AFP, it rose again (Figure 1(c)). In view of this unusual biochemical response for a GCT, the decision was made to obtain further tissue from the metastatic lesion in the parietal bone, chosen for its accessibility, whilst proceeding with definitive “VIP” chemotherapy from d12, namely 21-day cycles of etoposide (total 500 mg/m^2^), ifosfamide (total 6000 mg/m^2^), and cisplatin (total 100 mg/m^2^), delivered over 5 days, as described.^8^ A bleomycin-free approach was chosen in view of the mediastinal primary and presumed subsequent need for surgical resection after induction chemotherapy, with possible cardiopulmonary bypass and risk of respiratory failure with bleomycin, highlighted elsewhere.^8^

Parietal bone biopsy was performed just prior to cycle 2 VIP (d31), by which time the AFP had fallen to 967 kU/L (Figure 1(c)). CT reassessment at the same time demonstrated marked tumor volume reduction of the mediastinal mass (Figure 1(g)). However, there was interval increase in the known bone lesions, considered to potentially represent treatment-effect given the primary lesion response. Unfortunately, tissue from the parietal bone biopsy was again uninformative, H&E staining showed fragments of bone with abundant necrosis and fibrosis within the medullary cavity. Scattered within the fibrosis were crushed cells with high nuclear:cytoplasmic ratios; these cells had pleomorphic, hyperchromatic nuclei, and a rim of clear cytoplasm (Figure 1(h)). Immunohistochemistry demonstrated weak AFP positivity (Figure 1(i)). In addition, weak SALL4, patchy CD99, patchy CD117, diffuse Bcl-2, and focal EMA positivity was identified. CD3, CD79a, CD30, PLAP, Desmin, MyoD1, Glypican-3, OCT3/4, and WT1 were negative. There was no evidence of EWSR1 or SS18 gene rearrangement by FISH. Appearances were felt to be most in keeping with a NSGCT subtype of malignant GCT. However, a definitive diagnosis was not possible due to the small number of malignant cells and challenging morphology. Isochromosome 12p FISH testing was not possible due to the tiny amount of available biopsy tissue available and considerable crush artefact. There was ongoing concern regarding the biochemical and radiological response, with fluctuating AFP levels (Figure 1(c)), as well as a further increase in the size of the metastatic bone lesions. Moreover, serum miR-371a-3p testing for a malignant GCT diagnosis^12-14^ performed twice on multiple samples from presentation using current diagnostic criteria^15^ were all completely negative (Figure 1(j)). At this time, careful consideration was given regarding whether to change to second-line treatment; however, as there was some evidence of response clinically and biochemically (AFP), and radiologically in the primary tumor, treatment with VIP was continued (d51), whilst biopsies of both femoral head and vertebral lesions (again chosen for accessibility) were obtained under the same anesthetic, just prior to the final cycle 4 of VIP being delivered on d72.

**Figure 2.**
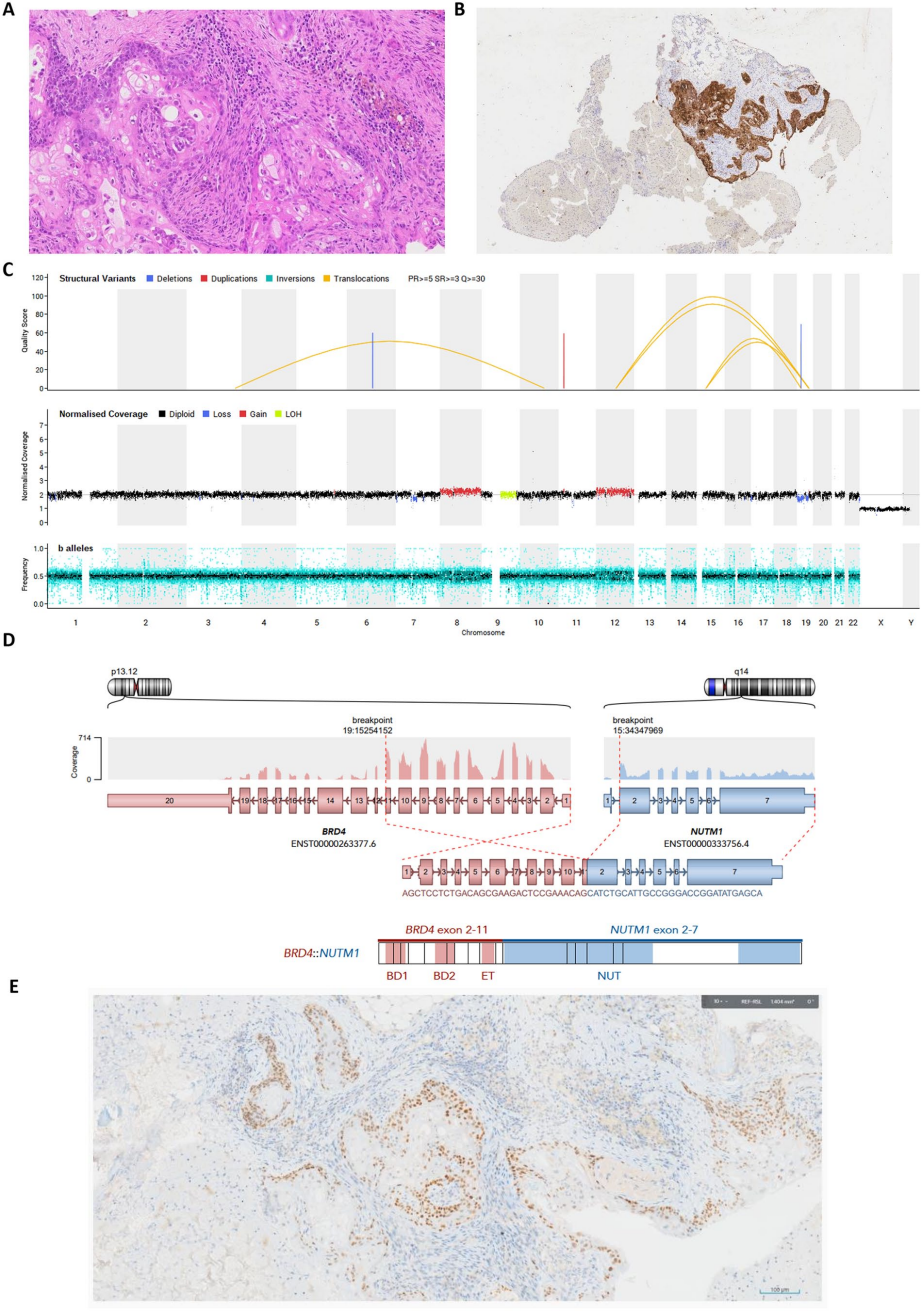
Further pathological, molecular, and whole genome sequencing (WGS) results of the NUT carcinoma case. (A and B) Pathology of the femoral head lesion biopsy: (A) H&E examination revealing multiple foci of immature squamous epithelium, occasionally closely associated with more basaloid, poorly-differentiated cells. Prominent bone remodeling with abundant osteoclasts was present, likely secondary to local tumor destruction and use of granulocyte colony stimulating factor (GCSF) treatment; (B) Immunohistochemistry revealed the poorly-differentiated cells to be positive for cytokeratins AE1/AE3. The cells were negative for PLAP, OCT3/4, and CD30, and in contrast to previous biopsies, this biopsy was negative for AFP, SALL4, and Glypican-3. (C) Global tumor mutational profile obtained through WGS performed on the tumor and paired normal sample, revealed a somatic translocation between chromosomes 15 and 19. Top track: structural variants; middle track: copy number; bottom track: b-allele frequency. Arcs between chromosomes 15 and 19 show the translocation, t(15;19)(q14;p13.12; arrow), resulting in the *BRD4::NUTM1* gene fusion. (D) *BRD4::NUTM1* gene fusion expression detected by RNA fusion analysis. The RNA breakpoints mapped to *BRD4* exon 11 (GRCh38 coordinates 19:15254152) and *NUTM1* exon 2 (GRCh38 coordinates 15:34347696), consistent with expression of an in-frame *BRD4::NUTM1* gene fusion. Key: BD1: Bromodomain 1; BD2: Bromodomain 2; ET: Bromodomain extra-terminal; NUT: NUT protein. No other pertinent somatic small variants or germline variants were detected, nor targetable variants. (E) NUTM1 immunohistochemistry on the parietal biopsy revealing positive staining.

As the patient was recovering from cycle 4 of chemotherapy, and 3 months following initial presentation, he developed worsening chest pain and a new palpable scalp mass. Serum AFP started to rise again (Figure 1(c)) and repeat CT confirmed radiological progression of both the mediastinal primary and metastatic bone lesions (Figure 1(k) and (l)). A femoral head lesion biopsy was performed, which contained cytokeratin-positive (AE1/AE3) poorly-differentiated cells with immature squamous epithelium, which, considering previous biopsies, was felt to best fit with a teratomatous element of a malignant NSGCT (Figure 2(a) and (b)); no evidence of viable tumor was observed in the vertebral lesion biopsy. Simultaneously, molecular results were returned from the parietal scalp biopsy, with WGS detecting a somatic translocation between chromosomes 15 and 19 (Figure 2(c)), resulting in a *BRD4::NUTM1* gene fusion demonstrated on RNA-based next-generation sequencing testing (Figure 2(d)), confirming NUT carcinoma. Retrospective NUTM1 immunohistochemistry on the parietal lesion confirmed positive staining (Figure 2(e)). Given the rapid clinical deterioration, and after discussion with the patient and family, no further active treatment was delivered, and the patient was managed with best supportive care and died 4 months following initial presentation.

## Discussion

This case highlights the need for maintaining a high index of suspicion to challenge diagnoses which have not been histologically and/or molecularly confirmed. Based on the picture of a mediastinal primary tumor with raised serum AFP and bone lesions in a young patient, the initial clinical diagnosis was thought to most likely represent a metastatic mediastinal malignant NSGCT. Whilst it is feasible to successfully treat patients with such tumors without recourse to biopsy, for example, Murray et al,^8^ there were features in this case, namely lack of pulmonary metastases and only modest serum AFP elevation relative to tumor bulk^16^ that meant the clinical team believed it imperative to pursue histological and molecular confirmation of the diagnosis upfront. However, attempts at histological assessment on the multiple biopsies ultimately obtained were challenging and non-diagnostic due to non-specific, undifferentiated morphology and poor cellularity with hemorrhage, acute inflammation, fibrosis, necrosis, and crush artefacts, commonly reported in NUT carcinoma.^3^ Further, the presumed GCT diagnosis was not excluded by the findings on initial thoracic and parietal biopsies; in particular, AFP, SALL4, and Glypican-3 expression is seen in many malignant GCT cases with yolk sac tumor (YST) components and have specifically been reported as highly sensitive and specific for mediastinal GCTs.^17^ However, to avoid misdiagnosis, it has been highlighted that an aberrant GCT-like immunophenotype (e.g., SALL4/AFP positivity) can arise in NUT carcinoma, as both diseases predominantly affect a young population, frequently involve the mediastinum, and can be associated with elevated serum AFP.^18^ Histologically, NUT carcinoma is typically composed of sheets and nests of monomorphic, small- to intermediate-sized undifferentiated cells. Tumor cells have evenly sized nuclei with irregular outlines, vesicular chromatin, and prominent nucleoli. There is a lack of nuclear molding and characteristically sheets of cells often show some separation between individual cells. Mitotic figures are readily identified and there is often necrosis. Abrupt keratinization is seen in around one-third of cases. Both primary and metastatic sites can show mesenchymal differentiation. NUTM1 antibody staining is positive in the vast majority of cases, showing a speckled nuclear pattern; however, confusingly, weak NUTM1 staining may also be seen in some GCTs. Cytokeratins are usually positive. There is frequent CD34 staining which may be misleading. Ki67 expression is high. In contrast, from a GCT perspective, YSTs can show a variety of histological patterns, including reticular, myxoid, macrocystic, solid, glandular, endodermal sinus, hepatitis, papillary, and sarcomatoid patterns, often observed in combination. AFP staining is frequently positive but often lacks sensitivity. SALL4 and pancytokeratins are diffusely positive. CDX2 and GATA3 are frequently positive, CD117 is variably positive, and CK7 is often negative or only focally positive.

Other NUT carcinoma case reports highlight the association of mediastinal tumors and raised serum AFP levels, mimicking malignant GCTs.^18-24^ These differentials remained the only 2 likely diagnoses based on the findings of a thoracic primary tumor with raised serum AFP. Thus, awareness of potentially misleading serum AFP elevations, along with the histological finding of poorly-/un-differentiated tumors lacking glandular differentiation, mandate testing for NUTM1 by immunohistochemistry.^21^ This case also highlights for the first time the clinical utility of negative serum miRNA (miR-371a-3p) testing in an NUT carcinoma case with elevated serum AFP levels. For absolute certainty, we ran the miR-371a-3p assay PCR^15^ multiple times, all of which were negative, making a malignant GCT diagnosis very unlikely.^12-14^

During treatment, due to the ongoing clinical suspicion that the presumed malignant GCT diagnosis was not correct, further biopsies from enlarging metastatic sites (parietal, femoral, and vertebral) were taken. Histologically, the working diagnosis (considering the reduction in size of the primary tumor) was that of a malignant GCT that had at diagnosis metastasized to bone, and which had subsequently undergone a ‘growing teratoma syndrome’ (GTS) like phenomenon.^25^ This is occasionally observed during treatment with chemotherapy in malignant NSGCTs, where the tumor differentiates into teratomatous elements. Such elements can show squamous differentiation with positive cytokeratin staining, as observed in NUT carcinoma.^3^ However, for a diagnosis of GTS (and thus malignant GCT originally), serum tumor markers such as AFP must have normalized, not the case here, which remained refractory, and a malignant GCT diagnosis was also not supported by the negative serum miR-371a-3p levels at presentation.

Whilst it is feasible that the lack of AFP response seen here can be observed in refractory GCT cases, typically AFP/HCG markers fall as expected in patients with mediastinal NSGCTs during intensive induction chemotherapy,^8^ and any progression generally occurs later. However, radiological responses to chemotherapy in mediastinal NSGCTs are often minimal.^8^ In contrast, the rapid reduction in size of the primary tumor with apparent progression of the bony metastatic lesions described here is consistent with our previous observations of patients with NUT carcinoma, along with the lack of appropriate AFP response to therapy.

The benefit of offering agnostic routine molecular testing for all cancer patients, including WGS, at our center^26,27^ is again demonstrated in this case, which revealed the translocation between chromosomes 15 and 19 and the functional *BRD4::NUTM1* fusion gene product on RNA sequencing, confirming NUT carcinoma. Despite the initial diagnosis as a malignant NSGCT, aggressive conventional chemotherapy treatment was delivered in a timely manner but was not successful. The combination of resection surgery, initial radiotherapy, and ifosfamide-based chemotherapy could prolong survival in some patients,^28,29^ but this approach is only appropriate for a minority of cases without extensive metastatic disease at presentation. Different potential targeted therapies are being studied, such as bromodomain inhibitors^30^ and histone deacetylase inhibition.^31^ Unfortunately, outcomes for patients with NUT carcinoma remain very poor^1^ and further research and novel treatment approaches are required to improve outcomes.

In summary, we highlight a rare case of a young patient presenting with a mediastinal primary tumor with widespread bone metastases and raised serum AFP level, initially presumptively diagnosed and treated as a malignant NSGCT. Negative serum miR-371a-3p testing made such a diagnosis highly unlikely, and histopathology was non-informative. Ultimately, agnostic molecular testing on biopsy material confirmed a *BRD4::NUTM1* gene fusion diagnostic of NUT carcinoma. Such cases should prompt early testing for NUTM1 rearrangement by immunohistochemistry or other molecular techniques.
